# Algunas aportaciones de la pandemia de COVID-19 al aprendizaje en salud pública, clínica y gestión

**DOI:** 10.23938/ASSN.1122

**Published:** 2025-04-30

**Authors:** Joan Carles March

**Affiliations:** Escuela Andaluza de Salud Pública Instituto de Investigación Biosanitaria ibs.GRANADA Granada España

La pandemia de COVID-19 ha sido una experiencia única de aprendizaje de la humanidad. Y ello ha generado que los países hayan necesitado llegar a un acuerdo histórico sobre pandemias para reforzar la colaboración internacional, la equidad y la resiliencia frente a futuras amenazas sanitarias mundiales^1^. Este ha sido uno de los aprendizajes más importantes de cara a nuevas pandemias, que requieren solidaridad para combatirlas. Así, el informe de evaluación de la gestión de la pandemia plantea que España debería reforzar su acción en los organismos internacionales para apoyar las actividades de prevención de la próxima pandemia^2^.

El conocimiento alrededor de la pandemia ha ido a un ritmo trepidante, con la rápida producción de vacunas, de fármacos y de tratamientos no farmacológicos, de test diagnósticos, así como de respuestas ante los problemas de salud mental, destacando lecciones valiosas para el futuro^3^. La colaboración e innovación científica para avanzar en el conocimiento del virus de la COVID-19 han permitido también la monitorización de aguas residuales que permite anticiparse a riesgos^4^. Estos avances tendrían que aprovecharse para responder mejor a patógenos futuros.

Además, se ha puesto de relieve el importante papel que puede jugar el uso de datos epidemiológicos integrados en tiempo real, la inteligencia epidemiológica que permite anticipar riesgos, monitorizar el impacto de la crisis y de las medidas de control y evaluar su eficacia con el desarrollo de renovados sistemas de información que sean capaces de proporcionar datos fiables en tiempo real sobre potenciales amenazas^5^^,^^6^. También hay que destacar otros aspectos como la globalización de los datos, que ha revolucionado la forma en que se utilizan para la toma de decisiones en salud pública, y la capacidad de gestión de los sistemas de salud, el avance en I+D, la capacidad tecnológica y de innovación -de la industria en general y de la farmacéutica en particular- con la perspectiva de producir vacunas para los próximos años, a lo que hay que añadir los contratos de compra anticipada, entre otras medidas^3^. La epidemiología digital, aparecida en los últimos años, impulsa también la disponibilidad de datos generados fuera del sistema sanitario, la mayor capacidad de computación y los avances en los métodos de análisis de datos, con la irrupción con fuerza de la inteligencia artificial^3^, con información útil para decidir cómo y dónde actuar. 

Destaca, por otra parte, la eclosión repentina de publicaciones antes de haber sido validadas, la escasa relevancia en España de la transferencia de conocimiento a la vida real, los profundos cambios en la gobernanza y regulación de la investigación biomédica, que necesitan revisarse en España, con cambios en los ensayos clínicos para adaptarse a las necesidades sobrevenidas, junto al papel de la transparencia y la comunicación como ingredientes del posible éxito o no de las políticas de salud^3^. 

Los sistemas sanitarios han sufrido consecuencias negativas; el informe del CES^7^ apunta a una clara agudización del problema de la accesibilidad al sistema que suponen las listas de espera, la saturación de la atención primaria y la necesidad de reforzar este nivel, el retraso de las citas y la postergación de pruebas y tratamientos en hospitales, con consecuencias en la salud de los pacientes. Es preocupante la situación de la salud mental en España y la insuficiencia de los dispositivos existentes para su correcta atención. Por otro lado, la crisis sanitaria por la COVID-19 no ha hecho sino acrecentar el reto de garantizar un nivel de gasto y un sistema de financiación que garanticen la adecuación de recursos para una prestación de servicios de calidad, adaptada a los requerimientos de una sociedad cada vez más exigente. La dura experiencia de la pandemia ha evidenciado asimismo la necesidad de profundizar en las garantías de los derechos del paciente, en un nuevo escenario donde las cuestiones relacionadas con la seguridad y la información en torno a los actos clínicos adquieren una nueva dimensión en materia de participación en el sistema^7^.

Una parte de los errores en la respuesta a la pandemia se debió a problemas preexistentes en el sistema sanitario, entre los que destacan: la distancia entre la salud pública y los niveles asistenciales, las deficiencias en los sistemas de vigilancia epidemiológica, unos recursos humanos estructuralmente infra-dimensionados para la actividad cotidiana de los servicios de salud pública, y la ausencia de un adecuado sistema de información a nivel nacional. Mención aparte merece la falta de protocolos previos en los centros sociosanitarios para personas mayores y otros colectivos vulnerables, y la limitada coordinación entre el sistema sanitario y los servicios sociales, que facilitó la tragedia vivida en las residencias de mayores^2^. Mención aparte merece la salud pública, cuya necesidad de reforzamiento es una evidencia^7^. 

Todo ello nos ha llevado a algunas lecciones que han surgido de la pandemia de COVID-19: inversión en salud pública e infraestructura de salud, contramedidas (médicas y no médicas), comunicación de riesgos y medidas de salud pública, e inversión en personas y alianzas^8^. A partir de la pandemia se plantea identificar a grupos de riesgo para orientar una toma rápida de decisiones, y su enlace con otros sectores (incluyendo la atención sanitaria y sociosanitaria). También establecer y reforzar las alianzas y colaboraciones con las administraciones públicas de sectores no sanitarios y con otros agentes para proteger la salud de las poblaciones, incluyendo actuaciones sobre la desinformación. Junto a ello, plantea contratar un personal altamente competente y diverso, con mayores capacidades en las diferentes áreas de la salud pública, así como la actualización de perfiles laborales (no solo salubristas, sino de comunicación, ciencias de datos, etc.) y una formación adaptada a las competencias necesarias en la salud pública del siglo XXI^6^.

Los aprendizajes de la pandemia han detectado aspectos que necesitan más investigación, como por qué un porcentaje de las personas afectadas han presentado COVID persistente en algún momento^9^^-^^11^. 

Otros estudios publicados en Anales del Sistema Sanitario de Navarra han aportado conocimientos sobre cómo las necesidades infantiles relacionadas con el estilo de vida se vieron afectadas por la falta de acceso a profesionales de la salud -y con mayor intensidad cuando los niños tenían una discapacidad-, enfrentándose a sentimientos de aislamiento por parte de los profesionales de la salud durante la COVID-19, lo que plantea líneas de mejora en estrategias dirigidas a promover la salud infantil^12^. También que los contactos domiciliarios mostraron un correcto nivel de conocimiento frente a la COVID-19 y sus medidas preventivas, así como que se transmitió correctamente que no todas las personas que enferman iban a desarrollar casos graves, que las personas con COVID-19 asintomáticas podían transmitir la infección y que, a pesar de estar vacunada, se podía transmitir la COVID-19, conocían la forma de transmisión, tenían conocimiento correcto sobre la higiene de manos y el uso de la mascarilla, información relevante para la toma de decisiones en caso de una nueva emergencia sanitaria^13^. Además, sabemos que el confinamiento domiciliario inicial y las medidas preventivas no farmacológicas contuvieron la circulación del SARS-CoV-2 hasta extenderse la vacunación, con la cual se logró una reducción decisiva en la gravedad y letalidad de la COVID-19. La presencia de anticuerpos indicativos de infección pasada ayudó a evitar infecciones por SARS-CoV-2^14^. 

Otros estudios publicados en esta revista han confirmado la utilidad terapéutica de los glucocorticoides para disminuir la mortalidad hospitalaria por COVID-19^15^. También destaca la prevalencia de los factores de riesgo de mortalidad hospitalaria por COVID-19, concordantes con los que se observan en otros registros. Se han identificado factores de riesgo clínico en pacientes vacunados que parecen afectar a la respuesta a algunas vacunas o medicamentos inmunosupresores, lo que puede ayudar a planificar el reclutamiento de los pacientes, a priorizar el refuerzo vacunal y a administrar futuros tratamientos específicos, realizando una clasificación de los pacientes según sus características a tener en cuenta. Asimismo, son necesarios nuevos estudios que permitan la validación de los factores de riesgo en cada escenario para obtener modelos predictivos para la COVID-19 que se adecúen a la virulencia del SARS-CoV-2, al estado inmunológico poblacional y las manifestaciones clínicas más frecuentes, para dar a la población el mejor tratamiento posible y mejorar con ello la asistencia hospitalaria^16^. La pandemia de COVID-19 ha supuesto una nueva llamada de atención sobre el potencial de la simulación como herramienta que mejora el trabajo en equipo, la coordinación entre profesionales sanitarios y el desenlace clínico de sus actividades^17^.

Como apunta el informe de la evaluación de la gestión de la pandemia^2^, es necesario generar un conjunto de medidas para mejorar la gestión de una pandemia: detección precoz, adecuada gestión de la crisis sanitaria con un comité científico-técnico, marco legal claro, estrategia de comunicación a la ciudadanía, protocolos de protección a los segmentos más vulnerables, atención primaria fortalecida con más salud comunitaria, reducir la carga de enfermedad crónica, mejora de las condiciones laborales del personal sanitario para protegerlos ante la ansiedad y el estrés y evitar el abandono de la profesión^18^, entre otras medidas, junto a la creación de la Agencia Estatal de Salud Pública^19^.
